# Evaluating the Efficacy of Teleophthalmology in Delivering Ophthalmic Care to Underserved Populations: A Literature Review

**DOI:** 10.3390/jcm12093161

**Published:** 2023-04-27

**Authors:** Joanna Dolar-Szczasny, Agnieszka Barańska, Robert Rejdak

**Affiliations:** 1 Chair and Department of General and Pediatric Ophthalmology, Medical University of Lublin, 20-079 Lublin, Poland; 2 Department of Medical Informatics and Statistics with E-Learning Laboratory, Medical University of Lublin, 20-090 Lublin, Poland; agnieszkabaranska@umlub.pl

**Keywords:** teleophthalmology, telemedicine, eyecare, rural populations, diabetic retinopathy, glaucoma, cataract, age-related macular degeneration

## Abstract

Technological advancement has brought commendable changes in medicine, advancing diagnosis, treatment, and interventions. Telemedicine has been adopted by various subspecialties including ophthalmology. Over the years, teleophthalmology has been implemented in various countries, and continuous progress is being made in this area. In underserved populations, due to socioeconomic factors, there is little or no access to healthcare facilities, and people are at higher risk of eye diseases and vision impairment. Transportation is the major hurdle for these people in obtaining access to eye care in the main hospitals. There is a dire need for accessible eye care for such populations, and teleophthalmology is the ray of hope for providing eye care facilities to underserved people. Numerous studies have reported the advantages of teleophthalmology for rural populations such as being cost-effective, timesaving, reliable, efficient, and satisfactory for patients. Although it is being practiced in urban populations, for rural populations, its benefits amplify. However, there are certain obstacles as well, such as the cost of equipment, lack of steady electricity and internet supply in rural areas, and the attitude of people in certain regions toward acceptance of teleophthalmology. In this review, we have discussed in detail eye health in rural populations, teleophthalmology, and its effectiveness in rural populations of different countries.

## 1. Introduction

As in every field, from science and education to communication and tourism, technological advancement has also transformed the face of medicine as we know it today. Over the years, incredible progress in surgical, diagnostic, and treatment procedures has been made. This progress has been achieved by improving efficiency, effectiveness, productivity, and innovation in different healthcare-related issues [1].

More simply, telemedicine, a combination of medicine and technology, is the use of information technology and telecommunication either in real time (synchronous) or stored and forwarded (asynchronous) to facilitate the assessment, diagnosis, and treatment of the remote patient [2]. Telemedicine has escalated the opportunities and possibilities to benefit medical education, communication, research, and healthcare delivery. It became progressively more prevalent in the early 1960s [3,4]. However, in that era, due to the high prices of audio and visual systems, combining the visual and audio data properly was not possible. Subsequently, recognition and acceptance of telemedicine were low against conventional medicine, which compromised the possibility and opportunity of substantial development during that era [5].

Then in the 1980s, with computers and digital development, a new digital era began. Eventually, the incorporation of these technologies facilitated the concomitant transmission of video, voice, and biometrics data, demonstrating significant progress. Moreover, the continued growth and advancement of communication technology led to the internet era with access to a cheaper global network of widespread technology. Furthermore, the capability to store large-scale, audio, image, and text information and to provide services has made robust advances in telemedicine in a short period [1].

Making healthcare accessible to the masses is the mainstay of telemedicine. It is achieved by preventing care delay due to the decrease in travel distance, thereby enabling primary and tertiary care accessible irrespective of the patient’s location [4].

As with other fields of medicine, ophthalmology has also witnessed the changes brought about by telemedicine that have been progressively adopted by various subspecialties. There are three types of teleophthalmology, namely: store and forward, real-time, and hybrid [6]. Studies have demonstrated the efficiency of teleophthalmology in terms of diagnosis and intervention for diseases where digital imaging is utilized for this purpose [7,8,9,10]. Apart from providing eye care, teleophthalmology has been proven helpful for disease screening by non-ophthalmologists. Most importantly, its significance in resource-limited regions of developing countries has been reported by studies and in rural and underserved populations worldwide [11,12,13,14,15]. However, there are various factors influencing the acceptance of teleophthalmology at a larger scale in various countries of the world [6,15,16,17]. We will discuss the factors influencing the acceptance of teleophthalmology in detail in a later section.

In this review, we will provide a detailed account of teleophthalmology and its current state in underserved populations of the world. The challenges, shortcomings, and benefits of teleophthalmology will be discussed in detail.

## 2. Status of Eye Care in Rural Population

Although healthcare is one of the fundamental needs of humans, due to poverty and economical imbalance, healthcare is not accessible to underserved populations in various regions of the world. Eye care remains the greatest neglected and unfulfilled healthcare need in major parts of the world. However, the conditions are more problematic in rural areas. Race, ethnicity, geographical location, socioeconomic status, existing disability, nutritional deficiency, lack of awareness, and non-availability and affordability of facilities are the factors affecting eye care access [18,19,20,21].

In the United States, the risk of chronic vision impairment increases among poor and less educated people, as their probability to visit an eye clinic is low [22,23]. This disparity also exists among children [24]. Likewise, homeless people also have higher rates of eye diseases, vision impairment, and uncorrected refractive error [25,26,27]. In countries such as Canada, which provide better health insurance coverage, homeless people there have better eye and vision care access as compared to the US, where limited eye access is associated with limited health insurance coverage. Thereby, health insurance coverage also has an impact on access to eye care [28,29,30,31,32].

At present, the universally existing eye care model is best demonstrated as reactive. Eye care providers are positioned on the sidelines, where patients are referred to them. Patients are referred to ophthalmologists by primary care physicians, emergency departments, long-term care facilities, and acute care hospitals.

## 3. Teleophthalmology: A Potential Solution

On the other hand, a more logical and efficient model of future eye care would be proactive and would assist in reducing the eye disease burden through the integration of eye care specialists and providers in primary care practices, acute care hospitals, emergency departments, and long-term care facilities where diagnostics, preventive, and therapeutic services are available at the site. Subsequently, this will improve access to eye care and early identification of asymptomatic patients, leading to a diagnosis of eye disease at an early stage on a population level and a reduction of a load of eye diseases on a larger scale. Nevertheless, implementing this model would increase the demand for several ophthalmologists according to the services needed. To cope with this need, we need to implement the use of technologies such as telemedicine [33].

## 4. Teleophthalmology and Its Effectiveness in Underserved Populations

Due to the growing evidence of reduced access to eye care and increase risk of eye disease in underserved populations, teleophthalmology programs for screening ocular diseases have been implemented at various levels for underserved people in various parts of the world. In this section, we will discuss the efforts and different programs that have been completed, their outcomes, and practices of teleophthalmology adopted in different parts of the world.

In New York City (NYC), real-time, mobile teleophthalmology services have already been launched and are accessible for the detection of eye disease in low socioeconomic and minority populations who are at risk. In a community-based launch, they screened 957 individuals in high-risk communities for eye diseases in the metro area of NYC between 2017 and 2018. Out of 957 screened persons, 458 (48%) were referred for ophthalmic evaluation. The optical assessment was performed using visual field, pachymetry, intraocular pressure, posterior segment optical coherence tomography, anterior segment optical coherence tomography, and nonmydriatic fundus photography. Glaucoma was identified in the highest number of participants 305 (32%), followed by narrow-angle 36 (14%), cataract 124 (13%), diabetic retinopathy (DR) 29 (3%), and macular degeneration 9 (1%), while 97 (10%) had other eye diseases [34]. This study indicated the feasibility and efficiency of teleophthalmology in increasing eye care access for the high-risk low socioeconomic population.

With time, adaptation to teleophthalmology has been witnessed in Iran [35,36,37,38,39,40,41,42,43]. A study conducted the comparison of tele-eye care with an on-site visit for diabetic retinopathy (DR) in diabetic patients. For teleophthalmology, a slit lamp was used to take images of the patient’s eyes. Stored images were then analyzed by retina specialists and general physicians. They found 90% specificity and 97% sensitivity in tele-eye care for the diagnosis of retinopathy. The sensitivity rate for retinopathy referral by retina specialists was 85% and 92%, while the sensitivity and specificity of their diagnoses of clinically significant macular edema (CSME) were calculated at 93% and 100%. For GPs, the sensitivity was 95% and 93%, and the specificity was 98%. In general, by implementing this teleophthalmology system, it was calculated that between 40% and 55% of unnecessary referrals could be avoided. They concluded that teleophthalmology was significantly accurate at diagnosis, and this can be utilized at a larger scale to save time, avoid unnecessary referrals, locate patients who are at a treatable stage, and increase access to specialists in rural areas where there are few or no specialists [44].

The rural areas in Western Australia have a shortage of workforce and facilities, a video-conferencing-based teleophthalmology program, Lions Outback Vision, has demonstrated significant advantages by enabling access to a greater number of people across this large area. These interventions reduce the number of outpatient visits to clinics, thereby allowing specialists to dedicate more time to surgeries. The most managed conditions were acute vision loss, abnormal retinal photographs, and red eye. These programs have achieved patient satisfaction as well [45,46,47,48,49,50]. A study conducted there to assess patient satisfaction for real-time video consultation reported that all the patients were satisfied with service, 69.1% were very satisfied and 24.5% were satisfied. Moreover, regression analysis did not show any relation with demographics [51].

In India, a country with a high burden of ocular diseases and vision impairment, a retrospective study of 5138 patients was conducted to evaluate the demographic data of patients who registered with an e-Sanjeevni OPD (outpatient department) for teleophthalmology services. They found that were more females (56%) than males (44%). The most commonly diagnosed eye condition was dry eyes (21%), followed by allergic conjunctivitis (18%), refractive error (15%), and cataract (14%), making up about 70% of diagnoses made by teleconsultations. The remaining diagnosed eye findings were diagnosed as blepharitis, stye, pterygium, nasolacrimal duct obstruction, and subconjunctival hemorrhage. Most patients (56.6%) were managed medically, and around 11.6% of the patients were referred for surgery [52].

The importance of teleophthalmology has been highlighted since the advent of the COVID-19 pandemic. During the lockdown, OPDs were shifted to clinics in various parts of the world. A study by Ravindran et al., was conducted to evaluate the experience of teleconsultation in a south Indian hospital during the COVID-19 pandemic lockdown. A total of 997 teleconsultations were made, and the most common (52.49%) queries were about general eye conditions such as watery eyes/redness/itching/irritation, then about medications (28.01%), followed by appointments (18.84%), and 0.64% were emergency needs for hospital due to sudden vision loss. Out of a total of 356 preterm babies screened, 16.01% were diagnosed with retinopathy of prematurity (ROP), three were given injections, and three required laser treatment. From the findings of this study, it has been demonstrated that this mode of teleophthalmology was highly helpful for eye care provided to patients [53]. Travel restrictions due to the COVID-19 outbreak led the Eye & Cornea Surgeons (ECS) clinic to initiate a telemedicine program that took a step toward developing a novel yet collaborative real-time virtual corneal clinic (VCC). A retrospective observational study by Siregar and colleagues found video real-time slit-lamp biomicroscopic examination (SLE), the most important clinical procedure during VCC, to provide maximum benefits [54].

Teleophthalmology-based DR screening in India has been conducted using fundus photographs. These photographs were captured using a hand-held nonmydriatic fundus camera that was present at diabetes management centers. Then, in the image reading center, L.V. Prasad Eye Institute Imaging Laboratory and Analysis Center (LILAC), images were qualified for DR by certified technicians, and in difficult cases, a specialist’s opinion was sought, thereby reducing the time for an ophthalmologist for screening these patients. In a pilot study of 229 diabetic subjects, the LILAC received fundus photographs of these subjects. Out of 229 screened subjects, 94 had treatable DR, while 32 subject images were ungradable (lens opacities, small pupil, etc.). It was concluded that with better internet facilities, teleophthalmology could be an effective approach for DR screening [55].

The Aravind Teleophthalmology Network in India is another example of teleophthalmology providing eye care access to the rural population. It is based on a mobile van fitted with a satellite for eye screening of diabetic patients in diabetologists’ clinics, camps, and hospitals. They have screened up to 20,080 patients in 74 camps conducted by the network. As reported by the Aravind Comprehensive Eye Survey Research Group, 10.6% of people in rural south India had DR, and only 6.7% of them had their eye examination previously [55,56,57].

In another study, the comparison was made between web-based teleretinal (TR) examination with in-person clinical assessment using slit-lamps for the diagnosis of DR and age-related macular degeneration (AMD) in diabetic patients in Kenya. All (*n* = 306) patients underwent both types of assessment. There was fair concordance for the diagnosis of AMD and DR between TR and in-person clinical assessment. Out of 612 photos from TR, 74 (12%) were not graded due to poor patient cooperation, media opacities, and unsatisfactory photos. Positive predictive values of TR for DR and AMD were 75% and 27.3%, respectively, as compared to slit-lamp clinical examination. The predictive value of negative TR diagnosis was 97.2% for DR and 98.1% for AMD. Thereby, these findings indicated that a teleretinal examination is an excellent approach for DR and AMD screening [58].

In rural areas of Nepal, patients with eye complaints go to the community eye center (CEC) that is nearest to their vicinity. The CECs are satellite sites of the TiIganga Institute of Ophthalmology (TIO), which is the only main eye care center located in Kathmandu. A study was conducted to implement the Paxos scope ophthalmology platform in four CECs of TIO for the characterization of referral patterns as demonstrated by routine examination and images from a specialized portable mobile device. In the CEC, an ophthalmic technician made referrals after a dilated eye examination. Photographs of posterior and anterior segments were captured using a Paxos Scope ophthalmic camera system attached to an iPod Touch sixth generation device. These images were then uploaded to a secure cloud database, which were then reviewed by an ophthalmologist in Kathmandu. The slit-lamp-based referral decision of the ophthalmic technician was compared with the recommendation of an ophthalmologist using transmitted images.

They found that ophthalmologists made 20% more referrals of the total screened subject (*n* = 364) than technicians. Moreover, 101 patients referred by technicians were confirmed as appropriate by the ophthalmologist in more than 97% of cases. However, they found that eight (2.8%) of those patients had variants of normal eye pathology. The authors reported that the use of teleophthalmology-based consultation with an ophthalmologist at TIO in Kathmandu would provide the required support to the staff at rural CECs for making a referral, identifying the patients who need ophthalmologists, and reducing the number of cases with missed diagnoses. Moreover, mobile device teleophthalmology could be an effective adjunct to the screening exams performed by ophthalmic technicians in CECs. Implementation of the Paxos teleophthalmology system in CECs improved the percentage of identified referable pathology [59]. Utilization of a mobile device-based ophthalmic camera improved the detection of ocular pathology, specifically the posterior segment. Subsequently, improving patient management and outcomes. Henceforth, mobile device-based ophthalmic cameras would be beneficial tools for teleophthalmology in rural and developing sites owing to their excellent durability, portability, affordability, and ability to take images of high quality [60,61].

A cross-sectional and multisite study was conducted by Sven-Erik Bursell et al., to investigate the prevalence of DR and diabetic macular edema (DME) using primary care teleophthalmology in diabetic patients of Native Americans (NA) and Alaskan Natives (AN). All the participants (*n* = 53,998) were recruited for retinal examination using clinically validated, non-mydriatic, retinal imaging and retinopathy assessment protocols for the identification of severity levels of proliferative diabetic retinopathy (PDR), non-proliferative diabetic retinopathy (NPDR), DME, and sight-threatening retinopathy (STR; a composite measure). The prevalence of PDR, NPDR, DME, and STR was 2.3%, 17.7%, 2.3%, and 4.2%, respectively. The lowest prevalence was in Alaska and highest in patients with HbA1c greater than or equal to 8%, using insulin, and having diabetes for more than ten years [62].

In a rural population in Spain, a study was conducted to investigate the prevalence of sight-threatening DR in diabetic patients in a rural area and the satisfaction level for teleophthalmology among patients and professionals. Out of a total of 752 diabetic patients who had undergone retinopathy examination, 114 were asked for the degree of their satisfaction through a questionnaire, and ten ophthalmologists were also asked about their satisfaction level with the teleophthalmology service they were part of. In total, 29.4% of the patients had sight-threatening DR, while 93.8% of the surveyed patients scored this activity as eight for satisfaction level, and all of them had an opinion to continue with this service. A total of 70% of the surveyed professionals scored the activity as eight for general satisfaction, 20% scored between seven and five, while one specialist did not respond. Furthermore, 90% of the professionals said that they would carry on with this activity, while the remaining 10% did not agree. The study concluded that teleophthalmology is helpful in the diagnosis of DR at earlier stages, is within access of the underserved, and is found satisfactory by patients and professionals both [63].

Zhijian et al., conducted a study to compare the cost of evaluation made by telemedicine-based digital retinal imaging with traditional fundus examination of DR in diabetic patients. A Canon CR-1 nonmydriatic fundus camera was used for the teleophthalmology of diabetic patients at the community center. The digital images acquired in the community office were stored in the EyePACS server network to be assessed by retinal specialists later at Yale Eye Center. Out of 611 patients, DR was diagnosed in 166 (27.2%) patients. For telemedicine-based assessment cost of training, devices, transportation, and annual charges, while for conventional examination cost staff labor, transportation, and current Medicaid reimbursement were used. Telemedicine-based digital retinal imaging assessment was found to be cheaper at USD 49.95 as compared to conventional fundus examination, which was USD 77.80 [64]. A summary of the above-mentioned studies is compiled in Table 1.

Establishing a teleophthalmology center in rural areas is a cost-effective option for providing remote ophthalmological care to patients and to address service inequalities. Implementation of virtual ophthalmology has shown promising results in lowering the occurrence of blindness. The growing body of literature has unleashed the potential of teleophthalmology concerning the delivery of cost-effective medical solutions for a number of eye diseases, particularly DR. It presents a promising solution to service disparities in areas lacking access to specialized medical services. By enhancing convenience, avoiding unnecessary visits and reducing travel cost, which are usually associated with non-teleophthalmology approach, virtual ophthalmology is presumed to be most beneficial for remote rural areas [65,66].

In China, a study compared the cost-effectiveness of teleophthalmology and traditional medicine for the screening of AMD and DR in people over the age of 50 years in rural and urban populations. They found that traditional medicine and teleophthalmology both were highly cost-effective in the combined screening of AMD and DR. They highlighted the importance of screening multiple blindness-causing conditions together in their clinical management. Moreover, they stressed the importance of early detection of AMD and DR with such screening programs and suggested making telemedicine an essential part of national eye examination programs [67].

In the past Markov model-based, cost-utility testing was performed to estimate the quality-adjusted life-years (QALYs) in distinct populations. A study conducted on prisoners by Aoki and coworkers showed the cost effectiveness of teleophthalmology (USD 16,514/18.73 QALYs) over teleophthalmology (USD 17,590/18.58 QALYs). Additionally, reduced incidence of blindness from 20.5 to 12.4% was seen when teleophthalmology was implemented [68]. In another study, Rachapelle and coworkers compared the cost-utility of telemedicine screening with no screening. They documented the approach as cost effective with USD 3134 per QALY gain, considering the cost of screening every five years. However, the cost-effectiveness was comparatively low for screening every two years [69]. Again, it is dependent on the establishment and the regular maintenance cost. The reduced travel and physician wait time has been documented for glaucoma patients going through virtual ophthalmology. Further, the associated cost was ~80% lower compared to in-person examination [70]. Li et al., also highlighted the cost-effectiveness of teleophthalmology and found it more convenient and accessible for disadvantaged and remote populations [64]. The “Canadian First Nations rural communities” inhabiting the underserved areas were studied by Kim and Driver. A direct savings of USD C56.34 was recorded per client using teleophthalmology in comparison with traditional ophthalmology. They suggested achieving much better results if teleophthalmology was offered on a constant basis [13]. A six-year prevalence study of DR also demonstrated a lower cost for telemedicine, generating savings of USD 752 for every examination in comparison to conventional in-clinic screening [71]. A recent study implemented telemedicine screening in a largely underserved urban/marginalized community residing in the Bronx, New York, employing non-mydriatic fundus cameras. The screening initiative was found to be cost-effective and unraveled significant societal benefit of 14.66 QALYs (~USD 35,471/QALY). Further, the initiative was effective in identifying many patients with DR and other eye disorders who may otherwise remain undiagnosed [72].

The world is continuously advancing in terms of technology, further upgrading telecommunication and subsequently optimizing teleophthalmology practices and improving cost-effectiveness. Nevertheless, the requirements for initial investment and maintenance and the potential to reduce medical costs via accurate diagnosis followed by treatment are ways toward enhanced working ability and improved quality of life, which cannot be disregarded. In brief, teleophthalmology is proposed to produce comparable outcomes as physical examination. This is also evident from the meta-analysis of Tan and coworkers, who documented the diagnostic accuracy of virtual ophthalmology compared to face-to-face consultation. Moreover, the medical literature proposes teleophthalmology as an efficient and safe alternative to conventional DR screening [73]. A common theme to the above-mentioned analysis however suggests the direct relation of patient uptake and the cost effectiveness, and one should identify the regional needs of integrating virtual approaches in the existing medical system to maximize the benefit gain. Further, urgent complaints presented to emergency rooms regarding ophthalmic events are usually misdiagnosed and are consequently mismanaged by non-ophthalmic personals. Telemedicine here provides an innovative approach capable of enhancing patient access to eye care for urgent ophthalmic complaints [74]. It is evident from the study of Ribeiro *and coworkers*, who highlighted the potential of teleophthalmology in emergent settings and remote regions with a sensitivity of 93% and specificity of 82% [75]. 

**Table 1:** Summary of studies reporting the effectiveness of teleophthalmology in providing eye care to rural populations in different regions of the world.

Authors	Objectives and Region	Key Findings
Al-Aswad, L.A. et al. [34]	Feasibility of mobile real-time ophthalmology for screening of eye diseases. USA	305 (32%) had glaucoma, (14%) had narrow-angle, 124 (13%) had cataracts, 29 had (3%) diabetic retinopathy, 9 (1%) had macular degeneration, and 97 (10%) had other eye diseases.
Keshvardoost, S. et al. [44]	Accuracy of teleophthalmology system in reducing unnecessary referrals. Iran	The sensitivity and specificity of diagnosis made through digital images were 90% and 97% for diabetic retinopathy while for clinically significant macular edema (CSME) were 93% and 100% compared with the face-to-face visit.
Host, B.K. et al. [51]	Assessment of patient satisfaction for real-time video consultation. Australia	69.1% reported the service as very satisfied while 24.5% reported as satisfied
Markan, A. et al. [52]	Retrospective analysis of demographic data of patients who availed an ophthalmology service (e-Sanjeevni) OPD. India	56% were females and 44% were males. Commonly diagnosed conditions, dry eyes followed by allergic (21%) conjunctivitis (18%), refractive error (15%), and cataract (14%).
Ravindran, M. et al. [53]	Evaluation of teleconsultation communicated in a hospital during COVID-19 lockdown. India	The majority of queries (58.93%) were directed to the department of the cornea, (16.26%) to the retina, (13.04%) to cataract, (10.14%) to glaucoma, and (1.61%) to pediatric ophthalmology. Out of 356 pre-term babies screened, 16.01% were diagnosed with retinopathy of prematurity (ROP).
Das, T. et al. [55].	Overview of the role of teleophthalmology for DR screening in rural populations. India	Fundus photographs of screened subjects were included. 94 had treatable DR, and images of 32 subjects were not graded due to poor quality
Nanji, K. et al. [58]	Comparison of teleretinal (TR) with clinical examination for assessment of DR and AMD in diabetic patients. Kenya	The positive predictive value of TR as compared to clinical examination for DR diagnosis was 75% and 27.3% for AMD. The negative predictive value of TR was 97.2% for DR diagnosis and 98.1% for AMD diagnosis.
Hong, K. et al. [59]	Comparison of referral recommendations after screening by an ophthalmic technologist at a remote site with an ophthalmologist investigating digital images obtained by the portable camera. Nepal	Ophthalmologists recommended 20% more referrals for total screened subjects than technicians. Ophthalmologists agreed with 97% of the referrals by technicians and found 2.8% as a variant of normal eye pathology.
Li, R. et al. [67]	Evaluation of cost-effectiveness of telemedicine and traditional medicine at the population level for diabetic retinopathy (DR) and age-related muscular degeneration (AMD). China	At the population level, combined screening of AMD and DR is cost-effective for people 50 years and more.
Bursell, S.-E. et al. [62]	Characterization of DR and diabetic macular edema (DME) in Native Americans (NA) and Alaskan natives (AN) using primary care teleophthalmology. USA	Prevalence of NPDR, PDR, DME, and STR among NA/AN patients was 17.7%, 2.3%, 2.3%, and 4.2%, respectively. The lowest prevalence was in Alaska while the highest was in patients with Hb1Ac greater than or equal to 8%
Martin, Y.V. et al. [63]	Evaluation of satisfaction level for ophthalmology in patients and doctors. Assessment of prevalence of DR in a diabetic patient in rural population using teleophthalmology. Spain	29.4% of patients had sight-threatening DR. 93.8% of patients and 70% of professionals were satisfied with teleophthalmology
Li, Z. et al. [64]	Comparison of cost-effectiveness of telemedicine-based digital retinal imaging assessment with traditional fundus examination of DR in diabetic patients. USA	Out of 611 patients, DR was identified in 166 (27.2%) patients. Telemedicine-based digital retinal imaging assessment was cheaper at USD 49.95 than conventional fundus examination, which was USD 77.80.
Fonda, S.J. et al. [76]	Estimation of the prevalence of diabetic macular edema (DME) and diabetic retinopathy (DR) in Native Americans and Alaska Natives (AI/AN) using macula-centered, non-mydriatic 200° field of view ultrawide field imaging (UWFI). USA	A high burden of diabetes involving complications was observed in the studied population. DME, DR, and any sight-threatening disease were found in 3.0, 28.6, and 3.0% of the individuals. DR was found to be associated with increasing age, while the chances of DME decreased with age. The observed results suggested the potential of UWFI in identification of early DR.
Chia, M.A. et al. [77]	Evaluation of the utility of the Melbourne Rapid Fields Screening (MRF-S) i-Pad module for detecting field defects in rural areas. Australia	Study shows the benefit of MRF-S in a non-metropolitan environment. This iPad module can identify patients with mild and moderate field defects while providing good user acceptance and short test duration.

To conclude, teleophthalmology is being practiced at different levels in different regions of the world, reflecting the status of healthcare needs, standards, and efforts in the respective region. The aforementioned studies have documented the effectiveness of teleophthalmology in providing eye care to underserved populations in different regions of the world. Thereby, we might conclude that teleophthalmology holds a promising future for eye care in underserved populations worldwide.

## 5. Artificial Intelligence, Recent Technological Advancements and Teleophthalmology: An Overview

The synergy and progress of digital innovation, i.e., communication and information technologies, have provided ophthalmologists with a supreme opportunity to adapt and implement new telehealth models. The Internet of Things (IoT), 5th generation telecommunication networks (5G), and artificial intelligence (AI) are some of the digital innovations that are continuously progressing and are now utilized in almost every aspect of our lives. AI-based algorithms and automatic robotics offer a potential alternative to hasten the implementation of virtual ophthalmology and to enhance its effectiveness by maximizing its utility [78].

Traditional diagnostic procedures for eye diseases rely on clinical assessment and image-capturing devices of different modes. The process is costly and time-consuming but at the same time can particularly be well suited to deep learning (DL) techniques. DL is a sub-branch of machine learning (ML), and its real-world applications to ophthalmic images, for instance visual fields and digital fundus photography, have been documented for automated screening of glaucoma [79], DR [80], retinopathy of prematurity (ROP) [81], and age-related macular degeneration (AMD) [82] with high accuracy.

A recent work by Li and coworkers demonstrated the development of a DL system, detecting common signs of DR and retinal hemorrhages using ~17 thousand UWF images. The system was found to have potential in detecting more DR patients with sensitivity and specificity of 96% in providing a retina view scope of the images that is 5× larger compared to traditional images [83,84]. Another recent example for ROP screening is the introduction of smartphone-based fundus imaging (SBFI), which has shown competitive results to typical contact fundus imaging [85]. 

A teleophthalmology system centered at Zhongshan Ophthalmic Center links 10 rural hospitals in Guangdong province. The system works in integration by combining fundus imaging and cloud-based and tablet applications, and provides a thorough examination of the eye, grading for glaucoma and DR, diagnosis of other eye related disorders and consequently the provision of treatment [86]. The new breakthroughs have been fostered by AI using supervised and unsupervised ML approaches to screen glaucoma. It is exemplified by a work of Li et al., who detected glaucomatous optic neuropathy by a DL system. The color fundus photographs were used to test the system, with sensitivity and specificity of 95.6 and 92.0%, respectively [87]. In a recent investigation, another DL model was developed utilizing spectral domain OCT images to automatize glaucoma detection [88]. Handheld portable non-mydriatic fundus cameras are another cost-effective approach, particularly in places with limited personnel and photographic equipment [89]. In the following years, Keel and coworkers analyzed retinal images and generated a DL model for neovascular AMD. The robust performance was evident from the sensitivity and specificity of 96.7 and 96.4%, respectively. The utilities are not the only limit here; the potential of virtual health consultancy has also been investigated to assess the suitability of corneal tissues for transplantation [90].

Clinical decision support systems (CDSS) are among the emerging tools within teleophthalmology. Inspired by the immense potential of CDSS, a recent and first study of its type was conducted to supplement the decision process, in particular in triage and referral. By utilizing precise diagnosis, streamlining the collection of information, and simultaneous elimination of obsolete paper-based consultations, the cloud-based CDSS has demonstrated higher levels of accuracy and urgency of consultations [91]. In brief, AI should be considered as a valuable tool to aid clinicians in supplementing their expertise, not a substituent of manpower. The field of ophthalmology can be revolutionized by the cutting-edge AI algorithms that make the process more target-oriented and that reduce workload and errors that otherwise may be caused by incomplete data integration. Here in the present review, we only discussed some of the research studies; however, for a more comprehensive clinical overview and evidence of recent AI applications in ophthalmology, the readers are suggested to refer to the works of Nuzzi and colleagues and Nikolaidou and coworkers [92,93].

Smartphones are now a part of everyone’s life; even unprivileged areas have extended social networks and are positively affected by healthcare systems. Although a slit lamp biomicroscope is a principal tool for ophthalmologists, in remote areas and underdeveloped regions, there could be limited access to slit lamps. If available, the cost is high for such settings, and such devices are not easy to transport and require experienced people to work with. Smartphones in this regard provide a user-friendly interface that is economical and saves time, thus bearing the potential to transform the clinician’s perspective toward assessing and managing eye disorders. Some of the devices that are documented for eye imaging are Peek [94,95], D eye [96], Cell Scope [97,98], and Eye Go [60], with Peek being highly efficient in performing a direct cataract test on a smartphone [94]. It is further endorsed from the work of Sanguansak and colleagues. Using various smartphone adaptors, images were acquired from the frontal corneal surface. At least 86 and 93% of the images were found readable and acceptable in terms of quality by an expert [99].

It is predicted that the growth of technology in the near future will enable patients to manage themselves using smartphones. Teleophthalmology assisted with smartphones can potentially prevent the disorders responsible for blindness. Tele-glucoma is another aspect where smart phones can be efficiently utilized to monitor, screen, diagnose and follow a patient with glaucoma. In relation to this, Russo and colleagues used an iPhone 5s attached to a D-EYE adaptor to reduce the reflection and simultaneous improvement in picture quality. They documented that under appropriate settings, smartphone imaging is nearly equivalent (cup/disc ratio) to slit lamp bio-microscopy, which is regarded as the gold standard in the field [100].

In addition to the above-mentioned applications of teleophthalmology, one should also think about the examination of visual functions, which nowadays can be tested remotely with the help of smartphones or tablets used by patients at home. This possibility of so-called home monitoring on smart devices with specially designed applications may involve both visual acuity and visual field examination. This method can be used to monitor treatment, especially in patients with AMD and glaucoma [77,101,102,103,104]. It may also be a good idea in patients who are at risk of developing such a disease or who are being observed for suspected disease. The problems may only be compliance and patient motivation in the long term, but the results of tests performed by the patient at home are characterized by high reliability and savings in time, equipment, and personnel. Such a solution can be part of home monitoring, which is considered one of the possibilities of telemedicine. In the future, it will probably be a common method in many centers. Another form of home monitoring currently being tested is the use of home tonometers to measure intraocular pressure (IOP) in patients being treated for glaucoma. Several types of devices are already available that allow for easy and reliable self-measurement of IOP. In many cases, this is an excellent solution to avoid frequent follow-up visits to the ophthalmologist’s office, but also to assess intraocular pressure fluctuations throughout the day and over several days [105,106]. The current problem may be the cost of equipment and thus availability in areas of lower economic status.

Briefly, teleophthalmology is a potential aspect of healthcare in underserved areas. It is efficient, effective in reducing false-positive referrals, and at the same time, saves time and money for both the patient and overall health system.

## 6. Advantages of Teleophthalmology

Teleophthalmology offers several advantages for eye care, particularly in rural and underserved areas.

- Efficiency and suitability: Teleophthalmology is efficient and well suited for eye diseases where digital imaging systems are beneficial for diagnosis and intervention [8,9,10].
- Short examination time: It allows for quicker examinations, enabling non-ophthalmologists to screen eye diseases effectively [9,10,11,15].
- Cost-effectiveness and accessibility: Teleophthalmology is cost-effective, accessible, reliable, and time-efficient, especially in rural areas where expert ophthalmologists may not be readily available [9,14,107,108,109,110].
- Reduced transportation costs: It helps reduce transportation costs for rural residents who would otherwise need to travel long distances to visit an eye specialist.
- Benefits for the elderly: Teleophthalmology is particularly advantageous for older individuals who have limited mobility and a higher risk of eye diseases [33].
- Increased access to ophthalmologists: Teleophthalmology allows a single ophthalmologist to serve a larger number of patients, saving time and reducing the duration of patient visits from 2–3 h to 20–30 min. This convenience can also help reduce the number of lost follow-ups [34].
- High satisfaction levels: Several studies have reported good satisfaction levels among patients and healthcare providers who use teleophthalmology services [111,112,113,114,115].

## 7. Limitations

Regardless of tremendous technological advancements and innovations, wider implementation of teleophthalmology is challenging. Teleophthalmology faces obstacles that are inherent in technology, such as access, communication process, quality, and security of the service. In teleophthalmology, it is expected that technology must store, compress, analyze, process, and visualize a large amount of data [1]. Although significant advancements have been made regarding the quality of images, poor-quality images are the major reason for referral to a retinal specialist with a frequency of 3–22%. This requires improvement in equipment and training of the staff [64,116,117].

Internet speed is one of the major limitations in its implementation, which is influenced by socioeconomic factors. For instance, in urban areas with well-structured facilities, the internet or its speed is not an issue where any fault can be resolved, and continuous advancements are readily adopted. On the contrary, the scenario is different in rural areas where there is already a lack of facilities, and internet speed and coverage are challenging [1,18,112].

Commonly used equipment in teleophthalmology includes a slit-lamp microscope, optical coherence tomography, and a fundus camera. These types of equipment have high cost. Furthermore, additionally required specific training for the staff and technicians to capture good-quality images for clinical assessment adds to the cost. In rural areas, there is a shortage of electricity as well, and installation of an uninterrupted power supply (UPS) to provide a steady supply of electricity is also an added cost.

Although there is a regulation system for tele-eye care, security and confidentiality of patient data (images) are also concerns [118,119].

In certain areas, there is less acceptance of telemedicine where technological advancements are not welcomed and where there is adherence to the mindset that face-to-face consultation is better than telemedicine [18]. In essence, teleophthalmology is opening exciting avenues for the provision of better eye care, but it is challenged by certain limitations that demand serious efforts.

## 8. Discussion

Teleophthalmology offers valuable services for rural populations around the world by addressing their eye health needs. Research has shown its benefits in terms of cost-effectiveness, efficiency, reliability, and patient satisfaction [82]. The primary focus of teleophthalmology applications has been on retinopathies, AMD, and glaucoma. Despite these advantages, teleophthalmology faces limitations that require attention from the scientific community to fully harness its potential [93]. The following recommendations could lead to improvements in teleophthalmology:

1. Large-scale government-funded screening programs would help identify patients with DR, AMD, and other ocular diseases at early stages.
2. Efforts should be made to familiarize and acclimate people with teleophthalmology [93].
3. Comprehensive training with uniform standards is necessary for health professionals in this field [120].
4. Consensus on image grading and agreement among health professionals is essential [121].
5. Establishing a regulatory body could ensure the provision of teleophthalmology equipment to countries with higher-risk underserved populations and limited government resources.
6. Further research is needed to develop policies regarding medico-legal liability for medical prescriptions and treating professionals, informed consent, and consultation payments [93].
7. Addressing the willingness to pay (WTP) for teleophthalmology in underserved populations is a concern; people should be educated about this [121].
8. Special training courses for staff and technicians responsible for capturing images and performing other tasks should be implemented.
9. Webinars and online sessions can help train ophthalmologists from countries with low medical literacy, with experts in the field providing guidance.

Our review has provided a comprehensive overview of the current state of teleophthalmology and its effectiveness for underserved populations across various regions of the world. We have also outlined the challenges involved in implementing teleophthalmology and offered recommendations to address these challenges, ultimately aiming to unlock the full benefits of ophthalmology. This information will be valuable for those working to advance and improve teleophthalmology.

## 9. Conclusions

There is little doubt that teleophthalmology is effective in providing eye care to underserved populations. Numerous studies have demonstrated its significance in terms of cost-effectiveness and accessibility of eye care while also achieving patient satisfaction. Although there is a possibility of poor-quality images, advancements in camera technology have proven their efficiency when compared to face-to-face examination and consultation. Despite certain obstacles and challenges, teleophthalmology has the potential to fulfill the need for eye care access in rural populations. However, further state-funded studies are needed to explore and harness its full potential.

## Data Availability

Not applicable.
